# Key differences between chronic inducible and spontaneous urticaria

**DOI:** 10.3389/falgy.2024.1487831

**Published:** 2024-10-17

**Authors:** Mojca Bizjak, Mitja Košnik

**Affiliations:** ^1^ Division of Allergy, University Clinic of Respiratory and Allergic Diseases Golnik, Golnik, Slovenia; ^2^ Faculty of Medicine, University of Maribor, Maribor, Slovenia; ^3^ Faculty of Medicine, University of Ljubljana, Ljubljana, Slovenia

**Keywords:** blood cell count, C-reactive protein, chronic inducible urticaria, chronic spontaneous urticaria, immunoglobulin E

## Abstract

**Introduction:**

The latest international EAACI/GA²LEN/EuroGuiDerm/APAAACI guideline for urticaria recommends limited laboratory testing for chronic spontaneous urticaria (CSU) and selective testing for only certain chronic inducible urticaria (CIndU) subtypes, though the rationale for these recommendations is poorly explained. This study aimed to improve the understanding of CIndU subtypes by comprehensively comparing their demographic, clinical, and laboratory characteristics with those of the better-characterized CSU.

**Methods:**

We conducted a retrospective analysis of 567 patients (median age 41 years, 67% female) diagnosed with CSU, symptomatic dermographism (SD), cold urticaria (ColdU), cholinergic urticaria (CholU), and delayed pressure urticaria (DPU).

**Results:**

Our findings revealed that patients with SD, ColdU, and CholU had lower levels of C-reactive protein (CRP), higher total serum immunoglobulin E (IgE) levels, and higher basophil counts compared to CSU patients. These subtypes also had distinct demographic and clinical features, such as a younger age of onset and a longer disease duration. In contrast, patients with DPU had significantly higher CRP levels and neutrophil counts compared to those with CSU.

**Discussion:**

These findings highlight the heterogeneity among chronic urticaria subtypes, suggesting that a tailored approach to laboratory testing may be more effective. The distinct immunological and clinical features observed in CIndU subtypes suggest a need for subtype-specific diagnostic and therapeutic guidelines.

## 1 Introduction

Chronic urticaria is a common disease with an estimated lifetime prevalence of 4.4% (1). However, significant challenges remain in its diagnosis and treatment. Chronic spontaneous urticaria (**CSU**) is characterized by recurrent wheals, angioedema, or both, occurring unpredictably and without specific triggers, as seen in chronic inducible urticaria (**CIndU**) (2, 3). The estimated point prevalence of CSU (0.02%–2.7%) is higher than that of the less researched CIndU (0.05%–1.5%) (4). Both can coexist in the same patient (5, 6). The most prevalent CIndU subtypes are symptomatic dermographism (**SD**), cold urticaria (**ColdU**), and cholinergic urticaria (**CholU**) (7, 8). Delayed pressure urticaria (**DPU**), an uncommon and poorly defined form of CIndU, is usually present in combination with CSU (4).

The pathogenesis of CSU is believed to involve mast cell degranulation triggered by pathogenic autoreactive immunoglobulin G (**IgG**) and/or immunoglobulin E (**IgE**) antibodies, complex interplay of mast cells and other skin-resident and infiltrating cells (e.g., T and B lymphocytes, monocytes, eosinophils, basophils, and neutrophils), as well as the activation of coagulation and complement systems (9–12). Autoimmune (type IIb) CSU, driven by autoreactive IgG against the high-affinity receptor for IgE (**Fc*ε*RI**) or IgE itself, is characterized by poor response to second-generation H_1_-antihistamines (**sgAHs**) and the monoclonal anti-IgE antibody omalizumab. It has also been associated with other autoimmune diseases, eosinopenia, basopenia, low total IgE, and elevated IgG against thyroid peroxidase (**IgG anti-TPO**) (13). Basic laboratory workup for CSU, including differential blood count (**DBC**), C-reactive protein (**CRP**), total serum IgE, and IgG anti-TPO, recommended by the latest international EAACI/GA^2^LEN/EuroGuiDerm/APAAACI urticaria guideline, may thus help identify type IIb CSU. In autoallergic (type I) CSU, mast cells are activated by autoallergens that crosslink autoreactive IgE antibodies bound to Fc*ε*RI. Type I CSU has been linked to normal or higher total IgE and a favorable response to omalizumab (14, 15). Autoallergic (type I) CSU is believed to be more common than autoimmune (type IIb) CSU, and growing evidence suggests that these two endotypes overlap in some patients (4, 16, 17).

MC activation and degranulation, with the subsequent release of histamine and other inflammatory mediators, is also a key driver of CIndU skin lesion development (18, 19), but the activating signals are not yet well defined (4). Autoallergic IgE-mediated mast cell activation has been suggested in SD, ColdU, and CholU through passive transfer experiments and/or the positive effect of omalizumab (4, 18, 20–22). So far, no autoantigen has been identified in CIndUs. Surface expression levels of the Fc*ε*RI on mast cells are positively regulated by IgE (23), and Fc*ε*RI expression has been found to be significantly higher in CIndU patients compared to healthy controls (24). The guideline recommends provocation tests for all CIndU subtypes, but laboratory tests (DBC, CRP) are only suggested as extended workup in SD and ColdU (2). The rationale for this selective recommendation is unclear.

CIndUs are long-persisting and challenging to manage diseases (18). This study aimed to enhance the understanding of the laboratory, demographic, and clinical features of CIndU subtypes by comparing them with the better-characterized CSU and with each other. This included assessing the practical value of determining automated complete blood count parameters (leukocytes with DBC, platelets, and erythrocytes) rather than just DBC in CSU, as well as complete blood count parameters, CRP, and total IgE in four CIndUs.

## 2 Materials and methods

### 2.1 Study design and population

This retrospective study included 567 unselected patients attending their initial consultation, aged 17–93 years (median [IQR]: 41 [30–53] years; 67% female) diagnosed with CSU and four subtypes of CIndU (SD, ColdU, CholU, and DPU). All patients were evaluated by the same urticaria specialist at a single tertiary care center during routine clinical practice. None had received prior omalizumab treatment. The study was approved by the Slovenian National Medical Ethics Committee (KME78/09/14). Informed consent was obtained from all participants. Chronic urticaria was diagnosed clinically and treated according to the established international guidelines. Each patient was followed up for at least three months and instructed to increase the dose of sgAHs according to the guidelines if needed. Exclusion criteria included suspected urticarial vasculitis (>48 h wheal duration and residual ecchymotic pigmentation), bradykinin-mediated angioedema, and glucocorticoid treatment within 7 days prior to blood withdrawal. Additionally, irregular uptake of sgAHs was an exclusion criterion for the analysis of response to these medications.

### 2.2 Laboratory evaluation

Blood samples were taken during active disease at the first visit, and the following routine laboratory tests were conducted at Clinic Golnik: CRP level (*n* = 549; Cobas 6000, Roche); automated complete blood count analysis with DBC (*n* = 567; Sysmex XN 3100, Sysmex); total serum IgE level (*n* = 288; Immulite 2000Xpi, Siemens); and IgG anti-TPO (*n* = 224; Cobas 6000, Roche). Ratios of neutrophil-to-lymphocyte (**NLR**) and platelet-to-lymphocyte (**PLR**) count were calculated. The cut-off values were as follows, based on previous studies, ≥5.0 mg/L for high CRP (25, 26), ≥2.5 for high NLR (27), <1.5 cells × 10^9 ^/L for lymphopenia (26, 28), <0.05 cells × 10^9 ^/L for eosinopenia (26, 29), <0.01 cells × 10^9 ^/L for basopenia (26, 29), <40 IU/ml for low total IgE (26, 30), and >100 IU/ml for high total IgE (31).

### 2.3 Clinical evaluation

Demographic and clinical parameters were collected. Response to sgAHs and omalizumab was assessed based on the Urticaria Control Test (**UCT**), a four-question patient-reported outcome measure used to assess disease control in both CSU and CIndU, with a recall period of four weeks. Controlled and uncontrolled chronic urticaria were defined as UCT score of 12–16 and 0–11, respectively (2, 32). Recidivant chronic urticaria was defined as a complete resolution lasting at least six months (despite exposure to relevant triggers in CIndU) in patients not receiving therapy, followed by a subsequent reappearance of signs and symptoms.

Provocation tests on the volar forearm, following protocols established in 2016 (7), were used to confirm SD, typical ColdU (**ColdU^T^**), and DPU. For assessment of SD, FricTest® was used in all patients. Local cold provocation tests with an ice cube melting in a small amount of water and TempTest® were conducted only when ColdU was suspected based on history. ColdU^T^ was defined by a positive local cold provocation over the stimulated area (33). In patients suspected to have DPU based on history, 5 kg rods with a 6.5 cm diameter lowered vertically on the forearm for 15 min were used. Swellings in DPU were considered as angioedema. CholU was diagnosed only in non-CSU patients based on a history of symmetrically distributed, short-lived itchy papular wheals repetitively induced by elevation of body temperature through physical activity (in all seasons, not solely in cold weather) and supported by mandatory photographs. Pulse-controlled ergometry was not routinely performed in an outpatient setting for technical reasons.

### 2.4 Statistical analysis

Data from electronic medical records and patient charts were collected and analyzed using IBM SPSS software version 25. Categorical variables, presented with frequencies and percentages, were analyzed using Fisher's exact test. The Kolmogorov–Smirnov test was used to assess normality of numerical variables. Since all variables were non-normally distributed, they were expressed as medians and interquartile ranges (IQR), and nonparametric tests were used. The Mann-Whitney U test was applied to compare two groups. The Spearman's rho rank correlation coefficient (*r*) was calculated to assess relationships between continuous variables and interpreted as weak (0.10–0.29), moderate (0.30–0.50), and strong (>0.50) (34). A *p*-value of less than 0.05 was considered statistically significant.

## 3 Results

### 3.1 Prevalence of CSU and CIndU subtypes

**Figure 1:**
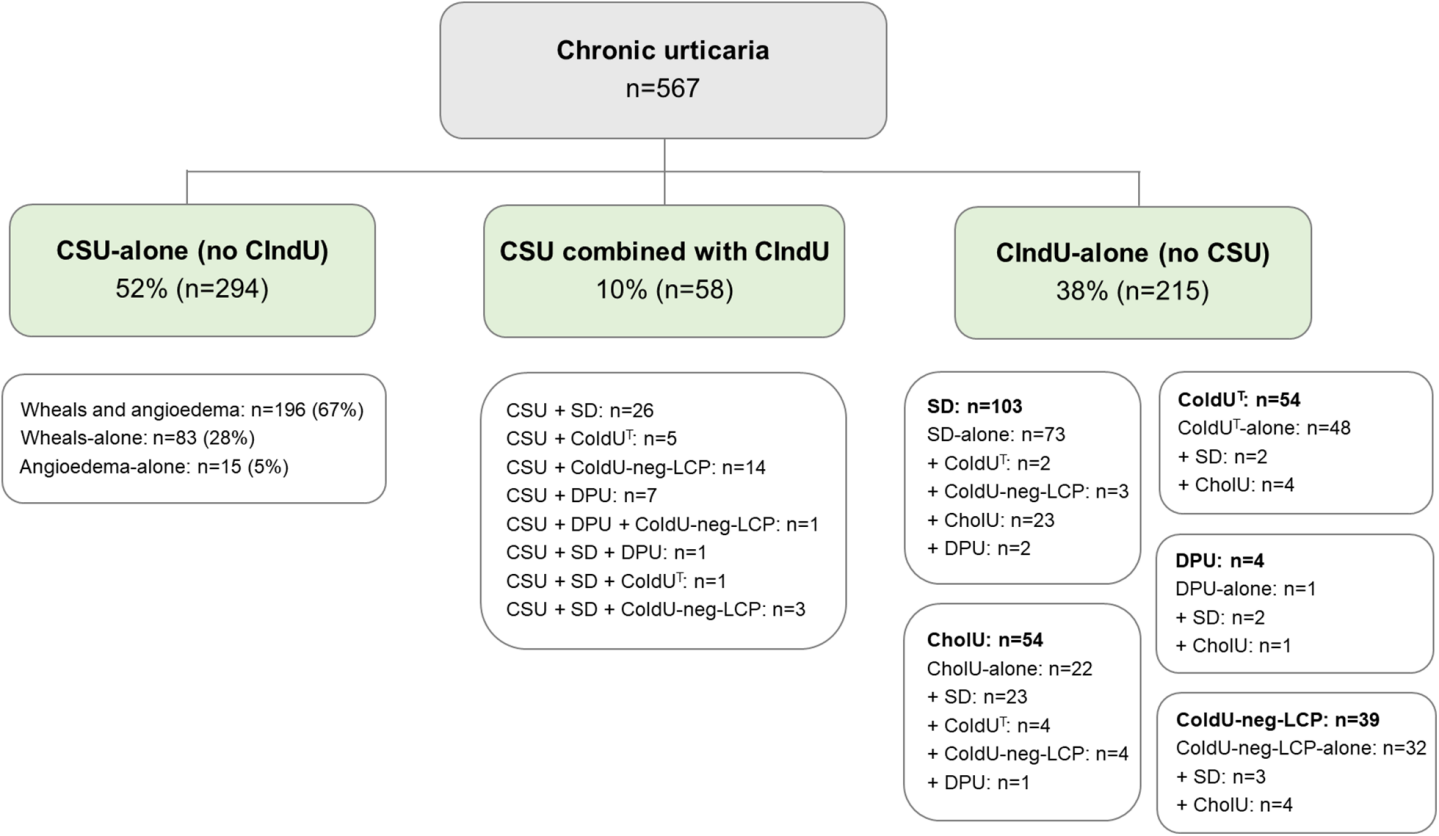
Distribution of chronic urticaria types and subtypes. Abbreviations: *CholU*, cholinergic urticaria during all seasons; *CIndU*, chronic inducible urticaria; *CIndU-alone*, chronic inducible urticaria without concomitant chronic spontaneous urticaria; *ColdU^T^*, typical cold urticaria; *ColdU-neg-LCP,* cold urticaria with negative local cold provocation tests; *CSU*, chronic spontaneous urticaria; *CSU-alone*, chronic spontaneous urticaria without concomitant chronic inducible urticaria; *DPU*, delayed pressure urticaria; *SD*, symptomatic dermographism.

CSU was more common than CIndU. Females were predominant in all studied types/subtypes of chronic urticaria, although this predominance was only marginal in CholU. CSU-alone was diagnosed in 52% (294/567), CSU combined with CIndU in 10% (58/567), and CIndU-alone in 38% (215/567) of patients. Among patients with CSU-alone, 67% (196/294) had both wheals and angioedema, 28% (83/294) had wheals-alone, and 5% (15/294) had angioedema-alone (Figure 1). Figure 1 also shows the prevalence of CIndU subtypes. The three most common CIndU subtypes (**3-CIndUs**) were SD, ColdU^T^, and CholU. DPU was rare. It was suspected and tested in 85 patients, but confirmed in only 13 of them. Nine patients had DPU combined with CSU, and four patients had DPU without CSU.

### 3.2 SD vs. CSU-alone

Patients with SD (*n* = 103), compared to those with CSU-alone (*n* = 294), had lower CRP (*p* < 0.001), neutrophils (*p* = 0.001), NLR (*p* < 0.001), and PLR (*p* = 0.002), a lower prevalence of eosinopenia (*p* = 0.005) and basopenia (*p* < 0.001), and higher basophils (*p* < 0.001) and total IgE (*p* = 0.015). SD patients also had a younger age at evaluation (*p* < 0.001) and disease onset (*p* < 0.001), longer disease duration (*p* < 0.001), lower rates of remission and recurrence (*p* < 0.001), a lower prevalence of skin angioedema (*p* < 0.001) and tongue angioedema (*p* = 0.002), shorter-lasting wheals (*p* < 0.001) and angioedema (*p* = 0.006), lower usage of glucocorticoids (*p* < 0.001), and fewer emergency department visits (*p* < 0.001). Among SD patients controlled with sgAHs (88%), 18% required a standard and 29% a 4-fold daily dose. SD patients less often needed a 4-fold sgAH dose for disease control compared to CSU-alone patients (*p* = 0.003) (Table 1).

**Table 1:** Characteristics of SD and ColdU^T^ compared to CSU.

Parameter	CSU *n* = 294	SD *n* = 103	ColdU^T^ *n* = 54	SD vs. CSU *p*-value	ColdU^T^ vs. CSU *p*-value
Demographics, course					
Female gender	194 (66.0)	67 (65.0)	35 (64.8)	0.904	0.877
Age (years)	45.0 (33.0–56.3)	37.0 (26.0–43.0)↓	39.5 (29.8–51.3)↓	**<0**.**001**	**0**.**042**
Age at disease onset (years)	42.5 (30.0–55.0)	34.0 (23.0–42.0)↓	33.0 (19.8–42.8)↓	**<0**.**001**	**<0**.**001**
Age ≤17 years	7 (2.4)	12 (11.7)↑	11 (20.4)↑	**0**.**001**	**<0**.**001**
Age 18–29 years	60 (20.4)	25 (24.3)	9 (16.7)	0.406	0.583
Age 30–39 years	56 (19.0)	34 (33.0)↑	16 (29.6)	**0**.**006**	0.099
Age 40–59 years	121 (41.2)	28 (27.2)↓	16 (29.6)	**0**.**013**	0.130
Age ≥60 years	50 (17.0)	4 (3.9)↓	2 (3.7)↓	**<0**.**001**	**0**.**011**
Disease duration (months)	6.0 (3.0–14.0)	12.0 (6.0–36.0)↑	24.0 (7.8–111.0)↑	**<0**.**001**	**<0**.**001**
Recidivant	49 (16.7)	1 (1.0)↓	^NA^	**<0**.**001**	^NA^
Max duration of wheals (h)	24.0 (6.0–24.0), *n* = 262	0.5 (0.5–1.0)↓	0.6 (0.5–1.0)↓	**<0**.**001**	**<0**.**001**
Max duration of angioedema (h)	24.0 (12.0–36.0), *n* = 205	3.0 (0.8–15.0)↓, *n* = 6	1.0 (0.5–1.0), *n* = 14↓	**0**.**006**	**<0**.**001**
Angioedema locations					
Skin, any location	209 (71.1)	7 (6.8)↓	17 (31.5)↓	**<0**.**001**	**<0**.**001**
Face	196 (66.7)	4 (3.9)↓	9 (16.7)↓	**<0**.**001**	**<0**.**001**
Upper extremities	60 (20.4)	4 (3.9)↓	5 (9.3)	**<0**.**001**	0.058
Lower extremities	61 (20.7)	4 (3.9)↓	1 (1.9)↓	**<0**.**001**	**<0**.**001**
Tongue	40 (13.6)	3 (2.9)↓	6 (11.1)	**0**.**002**	0.827
Oropharynx/larynx	1 (0.3)	0	11 (20.4)↑	1.000	**<0**.**001**
Treatment aspects					
Uncontrolled despite 4-fold sgAHs	37 (17.9), *n* = 207	8 (12.5), *n* = 64	7 (22.6), *n* = 31	0.345	0.619
Controlled with up to 4-fold sgAHs	170 (82.1), *n* = 207	56 (87.5), *n* = 64	24 (77.4), *n* = 31	0.345	0.619
Standard daily dose sufficient	21 (12.4), *n* = 170	10 (17.9), *n* = 56	3 (12.5), *n* = 24	0.370	1.000
2-fold dose needed	57 (33.5), *n* = 170	27 (48.2), *n* = 56	15 (62.5), *n* = 24↑	0.056	**0**.**012**
3-fold dose needed	5 (2.9), *n* = 170	3 (5.4), *n* = 56	0, *n* = 24	0.413	1.000
4-fold dose needed	87 (51.2), *n* = 170	16 (28.6), *n* = 56↓	6 (25.0), *n* = 24↓	**0**.**003**	**0**.**017**
Emergency department visit	54 (18.4)	0↓	2 (3.7)↓	**<0**.**001**	**0**.**004**
Glucocorticoids	63 (21.4)	1 (1.0)↓	0↓	**<0**.**001**	**<0**.**001**
Comorbidities					
Atopic disease	22 (7.5)	8 (7.8)	19 (35.2)↑	1.000	**<0**.**001**
Autoimmune disease	31 (10.5)	8 (7.8)	3 (5.6)	0.564	0.326
Laboratory characteristics					
CRP (mg/L)	1.9 (0.9–4.9), *n* = 285	1.1 (0.6–2.2), *n* = 98↓	1.3 (0.7–3.2)↓	**<0**.**001**	**0**.**037**
High (≥5 mg/L)	68 (23.9), *n* = 285	8 (8.2), *n* = 98↓	5 (9.3)↓	**0**.**001**	**0**.**018**
Neutrophils (×10^9 ^/L)	4.34 (3.46–5.38)	3.91 (3.20–4.60)↓	4.30 (3.47–5.02)	**0**.**001**	0.613
NLR	2.36 (1.78–3.17)	1.92 (1.56–2.61)↓	2.27 (1.65–2.80)	**<0**.**001**	0.280
High NLR (≥2.5)	129 (43.9)	29 (28.2)↓	23 (42.6)	**0**.**005**	0.883
Lymphocytes (×10^9 ^/L)	1.79 (1.49–2.18)	1.93 (1.54–2.38)↑	1.92 (1.49–2.49)	**0**.**049**	0.261
Lymphopenia (<1.5 × 10^9 ^/L)	76 (25.9)	22 (21.4)	14 (25.9)	0.426	1.000
Monocytes (×10^9 ^/L)	0.46 (0.37–0.59)	0.50 (0.42–0.62)	0.49 (0.39–0.60)	0.103	0.276
Eosinophils (×10^9 ^/L)	0.13 (0.07–0.20)	0.15 (0.09–0.22)	0.14 (0.08–0.19)	0.135	0.856
Eosinopenia (<0.05 × 10^9 ^/L)	45 (15.3)	5 (4.9)↓	9 (16.7)	**0**.**005**	0.838
Basophils (×10^9 ^/L)	0.03 (0.01–0.04)	0.03 (0.02–0.05)↑	0.04 (0.03–0.05)↑	**<0**.**001**	**<0**.**001**
Basopenia (<0.01 × 10^9 ^/L)	39 (13.3)	1 (1.0)↓	2 (3.7)	**<0**.**001**	0.063
Platelets (×10^9 ^/L)	262.5 (219.8–302.0)	245.0 (216.0–279.0)	264.5 (228.0–318.5)	0.051	0.294
PLR	143.6 (115.0–175.1)	124.5 (104.3–157.2)↓	140.8 (113.7–181.5)	**0**.**002**	0.893
Erythrocytes (×10^12 ^/L)	4.68 (4.34–4.95)	4.63 (4.37–4.93)	4.69 (4.40–4.93)	0.788	0.793
Total IgE (IU/ml)	61.0 (22.0–127.5), *n* = 125	83.5 (49.5–281.0), *n* = 34↑	98.5 (35.5–229.8), *n* = 50↑	**0**.**015**	**0**.**032**
Low (<40 IU/ml)	50 (40.0), *n* = 125	7 (20.6), *n* = 34	15 (30.0), *n* = 50	0.067	0.231
High (>100 IU/ml)	44 (35.2), *n* = 125	16 (47.1), *n* = 34	23 (46.0), *n* = 50	0.234	0.228
IgG anti-TPO (kU/L)	10.0 (8.0–15.3), *n* = 162	13.0 (9.0–19.0), *n* = 15	9.0 (9.0–12.0), *n* = 11	0.247	0.570
High (≥34 kU/L)	27 (16.7), *n* = 162	3 (20.0), *n* = 15	1 (9.1), *n* = 11	0.722	1.000

Categorical data are reported as *n* (i.e., number of patients with the outcome) and percentage (i.e., number of patients with the outcome/total number of patients in the group). Numerical data are reported as median (IQR). If data were not obtained in all patients, patient numbers are displayed as “*n*”. Fisher's Exact test was used for categorical variables and the Mann–Whitney *U* test for numerical variables. Statistically significant *p*-values are given in bold. Arrows (↑ and ↓) indicate a significantly higher or lower level/frequency of a parameter in a CIndU subtype compared to CSU.

Abbreviations: *ColdU^T^*, typical cold urticaria; *CRP*, C-reactive protein; *CSU*, chronic spontaneous urticaria; *IgE*, serum immunoglobulin E; *IgG anti-TPO*, immunoglobulin G against thyroid peroxidase; IQR*,* interquartile range; *NA*, not applicable; *NLR*, neutrophil-to-lymphocyte ratio; *PLR*, platelet-to-lymphocyte ratio; *SD,* symptomatic dermographism; *sgAHs,* second-generation H_1_-antihistamines.

### 3.3 Coldu^T^ vs. CSU-alone

Patients with ColdU^T^ (*n* = 54), compared to those with CSU-alone (*n* = 294), had lower CRP (*p* = 0.037), higher basophils (*p* < 0.001), and higher total IgE (*p* = 0.032). ColdU^T^ patients also had a younger age at evaluation (*p* = 0.042) and disease onset (*p* < 0.001), longer disease duration (*p* < 0.001), a lower prevalence of skin angioedema (*p* < 0.001), shorter-lasting wheals and angioedema (*p* < 0.001 for each), a higher prevalence of pharyngeal/laryngeal angioedema (*p* < 0.001), lower usage of glucocorticoids (*p* < 0.001), less frequent emergency department visits (*p* = 0.004), and a higher association with atopic diseases (*p* < 0.001). Among ColdU^T^ patients controlled with sgAHs (77%), 13% required a standard, while 25% needed a 4-fold daily dose. ColdU^T^ patients less often needed a 4-fold sgAH dose for disease control compared to CSU-alone patients (*p* = 0.017) (Table 1).

### 3.4 CholU vs. CSU-alone

Patients with CholU (*n* = 54), compared to those with CSU-alone (*n* = 294), had lower CRP (*p* < 0.001), neutrophils (*p* = 0.038), and NLR (*p* = 0.005); higher basophils (*p* = 0.005) and total IgE (*p* = 0.044); and a lower prevalence of basopenia (*p* = 0.002). CholU patients also had the earliest disease onset among all five studied chronic urticaria types. Compared to patients with CSU-alone (*n* = 294), CholU patients had a younger age at evaluation (*p* < 0.001) and disease onset (*p* < 0.001), longer disease duration (*p* < 0.001), lower rates of remission and recurrence (*p* = 0.002), a lower prevalence of skin angioedema (*p* < 0.001) and tongue angioedema (*p* = 0.002), shorter-lasting wheals (*p* < 0.001) and angioedema (*p* = 0.008), lower prevalence of autoimmune diseases (*p* = 0.041), lower usage of glucocorticoids (*p* < 0.001), and fewer emergency department visits (*p* = 0.001). Among CholU patients controlled with sgAHs (83%), 8% required a standard and 28% a 4-fold daily dose. CholU patients less often needed a 4-fold sgAH dose for disease control compared to CSU-alone patients (*p* = 0.034) (Table 2).

**Table 2:** Characteristics of CholU and DPU compared to CSU.

Parameter	CSU *n* = 294	CholU *n* = 54	DPU *n* = 13	CholU vs. CSU *p*-value	DPU vs. CSU *p*-value
Demographics, course					
Female gender	194 (66.0)	30 (55.6)	12 (92.3)	0.164	0.067
Age (years)	45.0 (33.0–56.3)	30.0 (21.0–38.0)↓	42.0 (37.0–54.5)	**<0**.**001**	0.711
Age at disease onset (years)	42.5 (30.0–55.0)	25.5 (19.0–36.3)↓	37.0 (27.0–46.5)	**<0**.**001**	0.169
Age ≤17 years	7 (2.4)	11 (20.4)↑	0	**<0**.**001**	1.000
Age 18–29 years	60 (20.4)	22 (40.7)↑	4 (30.8)	**0**.**003**	0.482
Age 30–39 years	56 (19.0)	13 (24.1)	6 (46.2)↑	0.457	**0**.**028**
Age 40–59 years	121 (41.2)	7 (13.0)↓	3 (23.1)	**<0**.**001**	0.254
Age ≥60 years	50 (17.0)	1 (1.9)↓	0	**0**.**002**	0.137
Disease duration (months)	6.0 (3.0–14.0)	21.0 (7.0–60.0)↑	24.0 (6.0–150.0)↑	**<0**.**001**	**0**.**013**
Recidivant	49 (16.7)	1 (1.9)↓	1 (7.7)	**0**.**002**	1.000
Max duration of wheals (h)	24.0 (6.0–24.0), *n* = 262	1.0 (0.5–1.0)↓, *n* = 52	^NA^	**<0**.**001**	^NA^
Max duration of angioedema (h)	24.0 (12.0–36.0), *n* = 205	2.0 (1.0–24.0)↓, *n* = 9	24.0 (18.0–48.0)	**0**.**008**	0.135
Angioedema locations					
Skin, any location	209 (71.1)	10 (18.5)↓	13 (100)	**<0**.**001**	0.023
Face	196 (66.7)	8 (14.8)↓	9 (69.2)	**<0**.**001**	1.000
Upper extremities	60 (20.4)	1 (1.9)↓	8 (61.5)↑	**<0**.**001**	**0**.**002**
Lower extremities	61 (20.7)	3 (5.6)↓	10 (76.9)↑	**0**.**007**	**<0**.**001**
Tongue	40 (13.6)	0↓	2 (15.4)	**0**.**002**	0.694
Oropharynx/larynx	1 (0.3)	1 (1.9)	1 (7.7)	0.287	0.083
Treatment aspects					
Uncontrolled despite 4-fold	37 (17.9), *n* = 207	5 (16.7), *n* = 30	3 (27.3), *n* = 11	1.000	0.428
Controlled with up to 4-fold	170 (82.1), *n* = 207	25 (83.3), *n* = 30	8 (72.7), *n* = 11	1.000	0.428
Standard daily dose sufficient	21 (12.4), *n* = 170	2 (8.0), *n* = 25	0, *n* = 8	0.744	0.599
2-fold dose needed	57 (33.5), *n* = 170	15 (60.0), *n* = 25↑	2 (25.0), *n* = 8	**0**.**014**	1.000
3-fold dose needed	5 (2.9), *n* = 170	1 (4.0), *n* = 25	1 (12.5), *n* = 8	0.566	0.244
4-fold dose needed	87 (51.2), *n* = 170	7 (28.0), *n* = 25↓	5 (62.5), *n* = 8	**0**.**034**	0.722
Emergency department visit	54 (18.4)	1 (1.9)↓	1 (7.7)	**0**.**001**	0.476
Glucocorticoids	63 (21.4)	0↓	1 (7.7)	**<0**.**001**	0.315
Comorbidities					
Atopic disease	22 (7.5)	6 (11.1)	1 (7.7)	0.411	1.000
Autoimmune disease	31 (10.5)	1 (1.9)↓	1 (7.7)	**0**.**041**	1.000
Laboratory characteristics					
CRP (mg/L)	1.9 (0.9–4.9), *n* = 285	0.7 (0.4–1.4), *n* = 52↓	6.7 (2.5–10.4)↑	**<0**.**001**	**0**.**008**
High (≥5 mg/L)	68 (23.9), *n* = 285	3 (5.8), *n* = 52↓	7 (53.8)↑	**0**.**003**	**0**.**023**
Neutrophils (×10^9 ^/L)	4.34 (3.46–5.38)	3.86 (3.29–4.91)↓	5.09 (4.63–6.17)↑	**0**.**038**	**0**.**031**
NLR	2.36 (1.78–3.17)	1.93 (1.59–2.64)↓	2.73 (2.36–3.29)	**0**.**005**	0.155
High NLR (≥2.5)	129 (43.9)	16 (29.6)	7 (53.8)	0.053	0.573
Lymphocytes (×10^9 ^/L)	1.79 (1.49–2.18)	1.87 (1.56–2.26)	1.81 (1.71–2.29)	0.213	0.428
Lymphopenia (<1.5 × 10^9 ^/L)	76 (25.9)	11 (20.4)	2 (15.4)	0.494	0.528
Monocytes (×10^9 ^/L)	0.46 (0.37–0.59)	0.51 (0.42–0.65)↑	0.53 (0.39–0.63)	**0**.**037**	0.278
Eosinophils (×10^9 ^/L)	0.13 (0.07–0.20)	0.13 (0.07–0.18)	0.13 (0.11–0.36)	0.734	0.236
Eosinopenia (<0.05 × 10^9 ^/L)	45 (15.3)	4 (7.4)	1 (7.7)	0.141	0.700
Basophils (×10^9 ^/L)	0.03 (0.01–0.04)	0.03 (0.02–0.05)↑	0.02 (0.01–0.05)	**0**.**005**	0.660
Basopenia (<0.01 × 10^9 ^/L)	39 (13.3)	0↓	2 (15.4)	**0**.**002**	0.687
Platelets (×10^9 ^/L)	262.5 (219.8–302.0)	254.5 (224.0–291.0)	283.0 (265.5–299.0)	0.671	0.135
PLR	143.6 (115.0–175.1)	132.3 (108.7–162.5)	147.2 (121.1–187.1)	0.087	0.650
Erythrocytes (×10^12 ^/L)	4.68 (4.34–4.95)	4.78 (4.42–5.10)	4.58 (4.47–4.82)	0.067	0.803
Total IgE (IU/ml)	61.0 (22.0–127.5), *n* = 125	98.0 (60.5–223.0), *n* = 17↑	117.0 (89.5–338.5), *n* = 5	**0**.**044**	0.094
Low (<40 IU/ml)	50 (40.0), *n* = 125	2 (11.8), *n* = 17↓	0, *n* = 5	**0**.**030**	0.156
High (>100 IU/ml)	44 (35.2), *n* = 125	7 (41.2), *n* = 17	4 (80.0), *n* = 5	0.788	0.062
IgG anti-TPO (kU/L)	10.0 (8.0–15.3), *n* = 162	12.0 (10.3–23.0), *n* = 10	9.0 (8.0–10.8), *n* = 8	0.292	0.358
High (≥34 kU/L)	27 (16.7), *n* = 162	2 (20.0), *n* = 10	0, *n* = 8	0.677	0.358

Categorical data are reported as *n* (i.e., number of patients with the outcome) and percentage (i.e., number of patients with the outcome/total number of patients in the group). Numerical data are reported as median (IQR). If data were not obtained in all patients, patient numbers are displayed as “*n*”. Fisher's Exact test was used for categorical variables and the Mann–Whitney *U* test for numerical variables. Statistically significant *p*-values are given in bold. Arrows (↑ and ↓) indicate a significantly higher or lower level/frequency of a parameter in a CIndU subtype compared to CSU.

Abbreviations: *CholU,* cholinergic urticaria; *CRP*, C-reactive protein; *CSU*, chronic spontaneous urticaria; *DPU*, delayed pressure urticaria; *IgE*, serum immunoglobulin E; *IgG anti-TPO*, immunoglobulin G against thyroid peroxidase; *NLR*, neutrophil-to-lymphocyte ratio; *PLR*, platelet-to-lymphocyte ratio; *sgAHs,* second-generation H_1_-antihistamines.

### 3.5 DPU vs. CSU-alone and other CIndUs

Compared to CSU-alone, DPU showed higher CRP (*p* = 0.008) and neutrophils (*p* = 0.031) and more commonly exhibited high CRP (*p* = 0.023). These parameters were also higher in DPU compared to those in 3-CIndUs. DPU (*n* = 13) had a higher female proportion (92%) than CSU-alone and 3-CIndUs. Clinical features of DPU were more similar to CSU-alone (*n* = 294) than 3-CIndUs. However, DPU patients, compared to CSU-alone patients, were more commonly aged 30–39 years at disease onset (*p* = 0.028) and had a longer disease duration (*p* = 0.013), a higher frequency of angioedema on the upper (*p* = 0.002) and lower extremities (*p* < 0.001), and a higher frequency of painful joints (31% [4/13] vs. 9% [26/294], *p* = 0.029). Tongue angioedema was common in both CSU-alone (14%) and DPU (15%). Among DPU patients controlled with sgAHs (73%), 63% required a 4-fold daily dose (Table 2).

### 3.6 Correlations between laboratory parameters

Table 3 provides insights into the relationships between immune blood cells, CRP, and total IgE according to Spearman's rho rank correlation coefficients. In CSU-alone patients, several significant positive correlations were observed: neutrophils with CRP, lymphocytes, monocytes, and platelets (*p* < 0.001 for each); lymphocytes with neutrophils, monocytes, eosinophils, basophils, and platelets (*p* < 0.001 for each); and monocytes with neutrophils (*p* < 0.001), lymphocytes (*p* < 0.001), eosinophils (*p* = 0.001), basophils (*p* < 0.001), and platelets (*p* = 0.019). Eosinophils were positively correlated with lymphocytes, monocytes, basophils, and total IgE (*p* < 0.001 for each), while basophils showed positive correlations with lymphocytes, monocytes, eosinophils, and total IgE (*p* < 0.001 for each) and an inverse correlation with CRP (*p* = 0.034). Platelets were positively correlated with neutrophils (*p* < 0.001), lymphocytes (*p* < 0.001), monocytes (*p* = 0.019), and CRP (*p* = 0.008). Additionally, Table 3 details the correlations between these blood parameters in four individual CIndU subtypes, highlighting variations across different forms of chronic urticaria.

**Table 3:** Spearman's rho rank correlation coefficients (*r*) between blood cell counts and CRP within CSU and cIndU subtypes.

		CRP (mg/L)	Neutrophils (×10^9 ^/L)	Lymphocytes (×10^9 ^/L)	Monocytes (×10^9 ^/L)	Eosinophils (×10^9 ^/L)	Basophils (×10^9 ^/L)	Platelets (×10^9 ^/L)
Neutrophils (×10^9 ^/L)								
CSU	*n* = 294	0.28***	NA	0.23***	0.29***	.	.	0.28***
SD	*n* = 103	.	NA	0.22*	0.36***	.	.	.
ColdU^T^	*n* = 54	.	NA	.	.	−0.35**	.	0.32*
CholU	*n* = 54	0.41**	NA	.	0.31*	.	.	.
DPU	*n* = 13	.	NA	.	.	.	.	.
Lymphocytes (×10^9 ^/L)								
CSU	*n* = 294	.	0.23***	NA	0.32***	0.29***	0.22***	0.28***
SD	*n* = 103	.	0.22*	NA	0.36***	0.21*	.	0.22*
ColdU^T^	*n* = 54	0.32*	.	NA	0.29*	.	.	0.30*
CholU	*n* = 54	.	.	NA	0.30*	0.38**	.	.
DPU	*n* = 13	.	.	NA	.	.	.	.
Monocytes (×10^9 ^/L)								
CSU	*n* = 294	.	0.29***	0.32***	NA	0.20**	0.25***	0.14*
SD	*n* = 103	.	0.36***	0.36***	NA	0.38***	0.43***	.
ColdU^T^	*n* = 54	.	.	0.29*	NA	.	.	.
CholU	*n* = 54	.	0.31*	0.30*	NA	0.44**	0.51***	.
DPU	*n* = 13	.	.	.	NA	.	.	.
Eosinophils (×10^9 ^/L)								
CSU	*n* = 294	.	.	0.29***	0.20**	NA	0.42***	.
SD	*n* = 103	.	.	0.21*	0.38***	NA	0.44***	.
ColdU^T^	*n* = 54	.	−0.35**	.	.	NA	0.36**	.
CholU	*n* = 54	0.28*	.	0.38**	0.44**	NA	0.34*	.
DPU	*n* = 13	.	.	.	.	NA	.	0.60*
Basophils (×10^9 ^/L)								
CSU	*n* = 294	−0.13*	.	0.22***	0.25***	0.42***	NA	.
SD	*n* = 103	.	.	.	0.43***	0.44***	NA	.
ColdU^T^	*n* = 54	0.30*	.	.	.	0.36**	NA	0.27*
CholU	*n* = 54	.	.	.	0.51***	0.34*	NA	0.33*
DPU	*n* = 13	.	.	.	.	.	NA	.
Platelets (×10^9 ^/L)								
CSU	*n* = 294	0.16**	0.28***	0.28***	0.14*	.	.	NA
SD	*n* = 103	0.23*	.	0.22*	.	.	.	NA
ColdU^T^	*n* = 54	0.32*	0.32*	0.30*	.	.	0.27*	NA
CholU	*n* = 54	0.40**	.	.	.	.	0.33*	NA
DPU	*n* = 13	.	.	.	.	0.60*	.	NA
Total IgE (IU/ml)								
CSU	*n* = 125	−0.18*	.	.	.	0.27**	0.33***	.
SD	*n* = 34	.	.	.	.	.	.	.
ColdU^T^	*n* = 50	.	.	0.30*	.	0.31*	.	.
CholU	*n* = 17	.	.	.	.	.	.	.
DPU	*n* = 5	.	.	.	.	.	.	.

*p*-values are indicated as follows: *for *p* < 0.05, **for *p* < 0.01, ***for *p* < 0.001, and a dot (.) for *p* ≥ 0.05.

Abbreviations: *CholU,* cholinergic urticaria; *ColdU^T^,* typical cold urticaria; *CRP,* C-reactive protein; *CSU*, chronic spontaneous urticaria; *CIndU*, chronic inducible urticaria; *DPU*, delayed pressure urticaria; *IgE,* serum immunoglobulin E; *NA*, not applicable; *SD,* symptomatic dermographism.

### 3.7 CRP, neutrophil counts, and NLR

Patients with CSU-alone had higher CRP (*p* < 0.001) and neutrophils (*p* = 0.001) compared to SD, higher CRP (*p* = 0.037) compared to ColdU^T^, higher CRP (*p* < 0.001) and neutrophils (*p* = 0.038) compared to CholU, and lower CRP (*p* = 0.008) and neutrophils (*p* = 0.031) compared to DPU (Tables 1, 2). In CSU-alone, high CRP [24% (68/285)] compared to normal CRP was linked to higher neutrophils (median [IQR]: 4.96 [4.19–6.10] vs. 4.15 [3.16–5.18] × 10^9 ^/L, *p* < 0.001), NLR (2.81 [2.13–3.68] vs. 2.27 [1.70–2.91], *p* < 0.001), and platelets (278.5 [228.3–315.3] vs. 254.0 [213.5–294.0] × 10^9 ^/L, *p* = 0.011), as well as a higher prevalence of painful joints (16% [11/68] vs. 7% [14/217], *p* = 0.024) (Supplementary Table S1). In CSU-alone, high NLR (44% [129/294]) compared to normal NLR was associated with higher CRP (2.5 [1.0–6.6] vs. 1.7 [0.7–4.0] mg/L, *p* = 0.011), lower eosinophils (0.10 [0.05–0.19] vs. 0.16 [0.09–0.22] × 10^9 ^/L, *p* < 0.001), eosinopenia (23% [30/129] vs. 9% [15/165], *p* = 0.001), and lower basophils (0.02 [0.01–0.04] vs. 0.03 [0.02–0.04] × 10^9 ^/L, *p* = 0.037) (Supplementary Table S2).

### 3.8 Features linked to poor response to treatment in CSU

Patients with uncontrolled CSU-alone despite a 4-fold daily dose of sgAHs [18% (37/207)] had higher CRP (median [IQR]: 3.9 [0.8–12.0] vs. 1.7 [0.9–4.5] mg/L, *p* = 0.026), lower eosinophils (0.10 [0.06–0.16] vs. 0.14 [0.07–0.22] × 10^9 ^/L, *p* = 0.033), lower basophils (0.01 [0–0.03] vs. 0.03 [0.02–0.04] × 10^9 ^/L, *p* < 0.001), and a higher frequency of basopenia (38% [14/37] vs. 11% [18/170], *p* < 0.001) compared with patients who were controlled with up to a 4-fold dose [82% (170/207)] (Supplementary Table S3).

Omalizumab was prescribed to 10% (*n* = 28) of CSU-alone patients. Nonresponders to omalizumab within 12 weeks [29% (8/28)] had lower monocytes (0.34 [0.26–0.43] vs. 0.45 [0.35–0.71] × 10^9 ^/L, *p* = 0.032) and eosinophils (0.10 [0.07–0.11] vs. 0.17 [0.09–0.24] × 10^9 ^/L, *p* = 0.049), a higher frequency of basopenia (63% [5/8] vs. 15% [3/20], *p* = 0.022), and higher PLR (188.6 [161.8–217.9] vs. 144.3 [119.2–165.2], *p* = 0.006) than patients who were controlled within 12 weeks (71% [20/28]). Late responders to omalizumab (by week 12; 45% [9/20]) were more commonly female (89% [8/9] vs. 36% [4/11], *p* = 0.028) and had higher CRP (4.6 [1.6-8.7] vs. 0.9 [0.5–2.2] mg/L, *p* = 0.044) and monocytes (0.74 [0.41–0.87] vs. 0.43 [0.33–0.52] × 10^9 ^/L, *p* = 0.020) than early responders (by week 4; 55% [11/20]) (Supplementary Table S3).

### 3.9 CSU combined with CIndU vs. CIndU-alone

Patients with concomitant CSU and CIndU (*n* = 58), compared to CIndU-alone patients (*n* = 215) (Figure 1), were older (median [IQR]: 39.5 [30.8–56.8] vs. 37.0 [26.0–45.0] years, *p* = 0.009), had a higher frequency of skin angioedema (48% [28/58] vs. 20% [43/215], *p* < 0.001), had a longer maximal duration of skin angioedema (18.0 [6.3–24.0] vs. 1.0 [1.0–5.0] hours, *p* < 0.001), a higher frequency of glucocorticoid use (9% [5/58] vs. 0.5% [1/215], *p* = 0.002) and omalizumab therapy (14% [8/58] vs. 5% [10/215], *p* = 0.031), lower basophils (0.03 [0.02–0.05] vs. 0.05 [0.03–0.06] × 10^9^ /L, *p* = 0.021), and higher CRP (2.70 [0.70–7.70] vs. 1.0 [0.6–2.3] mg/L, *p* < 0.001).

## 4 Discussion

This study examined a large cohort of 567 patients and identified significant differences in demographic, clinical, and laboratory parameters between four CIndU subtypes and CSU, which have the potential to enhance our understanding of chronic urticaria and guide future studies.

Our findings are consistent with previous research on demographic features: CIndU is less prevalent than CSU (4); SD is the most common CIndU subtype (6, 8); both CIndU and CSU are more prevalent in females (35); patients with 3-CIndUs are younger and have a later disease onset than those with CSU (4); CIndUs tend to have a longer duration before evaluation (36), possibly due to a perception of lesser severity leading to delayed specialist referrals; and individual wheals in 3-CIndUs are shorter lasting than in CSU (4, 36).

The recurrence rate for CSU in our study (17%) is comparable to the 21% observed by Toubi and Vadasz (37). We also confirmed that concomitant CSU and CIndU, compared to CIndU alone, are associated with older age, more frequent skin angioedema, and increased glucocorticoid use (6).

Our study supports current knowledge that in CSU, angioedema most commonly affects the face, less frequently the tongue, and rarely the oropharynx or larynx (38). Involvement of the oropharynx or larynx is more common in ColdU^T^, as previously described (39).

Complex correlations between blood leukocyte types in CSU, and to a lesser extent in CIndUs, suggest different immunological profiles. These correlations are difficult to interpret as many may not be specific to CSU. Nonetheless, they support the role of cellular infiltrates in CSU pathology (10). This contrasts with the minimal perivascular infiltrate seen in most CIndUs, except for DPU, where eosinophilic infiltration is noted (19). Neutrophil infiltration in CSU wheals during early phases may contribute to its pathogenesis, with prominent infiltration linked to therapy resistance (10).

NLR and CRP, markers of systemic inflammatory response (40), were elevated in CSU and DPU but not in 3-CIndUs, reinforcing the understanding of CSU as an immune-mediated chronic, systemic inflammatory disease (41, 42). DPU had even higher CRP levels compared to CSU and 3-CIndUs. High NLR (≥2.5) indicates chronic low-grade systemic inflammation (43). In our study, it was associated with higher CRP, lower eosinophils and eosinopenia, and lower basophils, but not basopenia. This suggests that the observed eosinopenia may be linked to systemic inflammation. Higher NLR and neutrophils have been associated with a lower remission rate in pediatric CSU patients (44), and some studies (40, 45, 46), but not all (47), have reported a decline in NLR with omalizumab treatment.

High CRP levels have been previously reported in CSU (25, 48, 49). CRP elevation may result from mast cell activation and subsequent inflammation (50). However, CRP is nonspecific and can be elevated in various diseases, including chronic infections and autoimmune disorders often seen with CSU (49), though levels are generally lower in CSU (51). Our study found high CRP in 24% of CSU patients, compared to 31% reported by Kolkhir et al. (25) using the same cutoff. Both studies found comparable median CRP levels in patients with high CRP; positive correlations between CRP, neutrophils, and platelets; an inverse correlation between CRP and basophils; a link between high CRP and higher neutrophils and platelets; and higher CRP levels in non-responders to sgAHs.

Our results highlight the challenge of effectively managing chronic urticaria with sgAHs. Standard-dose sgAHs controlled only 12% of CSU patients, lower than the 39% reported in a meta-analysis (52). Similarly, only 8%–18% of 3-CIndUs patients were controlled with standard doses. A fourfold daily sgAH dose was significantly more often needed for control in CSU than 3-CIndUs. Many CSU patients also visited emergency departments.

Consistent with previous reports, poor response to sgAHs in CSU was linked to lower eosinophils, and poor response to omalizumab was associated with lower eosinophils and basopenia (29). In our CSU patients, poor response to omalizumab was also linked to lower monocytes and higher PLR, which, to the best of our knowledge, is a novel finding.

The prevalence of autoimmune diseases in our CSU patients (11%) is higher than in the general population (≤1%), consistent with a systematic review (53). A genome-wide association study found a genetic overlap between CSU and autoimmune diseases, but not atopic diseases (54). The latter were also uncommon in our CSU patients. We found a higher prevalence of atopic diseases in ColdU^T^ and high total IgE (35% and 46%, respectively) compared to Neittaanmäki (25% and 30%, respectively) (55). The link between atopy and ColdU remains unclear (56). It is also believed that atopic predisposition and CholU are associated (57), but only 11% of CholU patients in our study had atopic diseases.

This study benefits from a large sample size and evaluation by a single physician, which ensures data consistency but may introduce bias. Additional limitations include the retrospective design, potential inaccuracies in automated blood count analysis, and the lack of age- and sex-matched healthy controls. The absence of pulse-controlled ergometry for CholU diagnosis and the small number of patients treated with omalizumab further limit generalizability and statistical power. The interpretation of higher total IgE levels in 3-CIndUs compared to CSU should be approached with caution due to potential variability related to atopic conditions, gender, and age (58), which were not precisely analyzed.

Our study offers new insights into the immunological and clinical heterogeneity of chronic urticaria and highlights the need for subtype-specific urticaria guidelines, particularly for CIndUs, to address the unique challenges in managing these conditions.

## Data Availability

The datasets presented in this article are not readily available because of privacy or ethical restrictions. Requests to access the datasets should be directed to mojca.bizjak@klinika-golnik.si.

## References

[1] Fricke J Avila G Keller T Weller K Lau S Maurer M Prevalence of chronic urticaria in children and adults across the globe: systematic review with meta-analysis. Allergy. (2020) 75:423–32. 10.1111/all.14037 31494963

[2] Zuberbier T Abdul Latiff AH Abuzakouk M Aquilina S Asero R Baker D The international EAACI/GA(2)LEN/EuroGuiDerm/APAAACI guideline for the definition, classification, diagnosis, and management of urticaria. Allergy. (2022) 77:734–66. 10.1111/all.15090 34536239

[3] Buttgereit T Vera C Aulenbacher F Church MK Hawro T Asero R Patients with chronic spontaneous urticaria who have wheals, angioedema, or both, differ demographically, clinically, and in response to treatment-results from CURE. J Allergy Clin Immunol Pract. (2023) 11:3515–25.e4. 10.1016/j.jaip.2023.08.020 37604426

[4] Kolkhir P Gimenez-Arnau AM Kulthanan K Peter J Metz M Maurer M. Urticaria. Nat Rev Dis Primers. (2022) 8:61. 10.1038/s41572-022-00389-z 36109590

[5] Weller K Gimenez-Arnau A Grattan C Asero R Mathelier-Fusade P Bizjak M The chronic urticaria registry: rationale, methods and initial implementation. J Eur Acad Dermatol Venereol. (2021) 35:721–9. 10.1111/jdv.16947 32946615

[6] Ornek Ozdemir S Kuteyla Can P Degirmentepe EN Cure K Singer R Kocaturk E. A comparative analysis of chronic inducible urticaria in 423 patients: clinical and laboratory features and comorbid conditions. J Eur Acad Dermatol Venereol. (2024) 38:513–20. 10.1111/jdv.19637 37991240

[7] Magerl M Altrichter S Borzova E Gimenez-Arnau A Grattan CE Lawlor F The definition, diagnostic testing, and management of chronic inducible urticarias—the EAACI/GA(2) LEN/EDF/UNEV consensus recommendations 2016 update and revision. Allergy. (2016) 71:780–802. 10.1111/all.12884 26991006

[8] Maurer M Fluhr JW Khan DA. How to approach chronic inducible urticaria. J Allergy Clin Immunol Pract. (2018) 6:1119–30. 10.1016/j.jaip.2018.03.007 30033913

[9] Kaplan A Lebwohl M Gimenez-Arnau AM Hide M Armstrong AW Maurer M. Chronic spontaneous urticaria: focus on pathophysiology to unlock treatment advances. Allergy. (2023) 78:389–401. 10.1111/all.15603 36448493

[10] Gimenez-Arnau AM DeMontojoye L Asero R Cugno M Kulthanan K Yanase Y The pathogenesis of chronic spontaneous urticaria: the role of infiltrating cells. J Allergy Clin Immunol Pract. (2021) 9:2195–208. 10.1016/j.jaip.2021.03.033 33823316

[11] Koren A Dejanović L Rijavec M Kopač P Bizjak M Zidarn M Autoimmune mast cell activation test as a diagnostic tool in chronic spontaneous urticaria. Int J Mol Sci. (2024) 25:9281. 10.3390/ijms25179281 39273229 PMC11395619

[12] Rijavec M Kosnik M Koren A Kopac P Selb J Vantur R A very low number of circulating basophils is predictive of a poor response to omalizumab in chronic spontaneous urticaria. Allergy. (2021) 76:1254–7. 10.1111/all.14577 32876979

[13] Kolkhir P Munoz M Asero R Ferrer M Kocaturk E Metz M Autoimmune chronic spontaneous urticaria. J Allergy Clin Immunol. (2022) 149:1819–31. 10.1016/j.jaci.2022.04.010 35667749

[14] Maurer M Eyerich K Eyerich S Ferrer M Gutermuth J Hartmann K Urticaria: collegium internationale allergologicum (CIA) update 2020. Int Arch Allergy Immunol. (2020) 181:321–33. 10.1159/000507218 32224621 PMC7265766

[15] Asero R Ferrer M Kocaturk E Maurer M. Chronic spontaneous urticaria: the role and relevance of autoreactivity, autoimmunity, and autoallergy. J Allergy Clin Immunol Pract. (2023) 11:2302–8. 10.1016/j.jaip.2023.02.022 36868473

[16] Elieh-Ali-Komi D Metz M Kolkhir P Kocaturk E Scheffel J Frischbutter S Chronic urticaria and the pathogenic role of mast cells. Allergol Int. (2023) 72:359–68. 10.1016/j.alit.2023.05.003 37210251

[17] Asero R Marzano AV Ferrucci S Lorini M Carbonelli V Cugno M. Co-occurrence of IgE and IgG autoantibodies in patients with chronic spontaneous urticaria. Clin Exp Immunol. (2020) 200:242–9. 10.1111/cei.13428 32115683 PMC7231996

[18] Munoz M Kiefer LA Pereira MP Bizjak M Maurer M. New insights into chronic inducible urticaria. Curr Allergy Asthma Rep. (2024) 24:457–69. 10.1007/s11882-024-01160-y 39028396 PMC11297124

[19] Church MK Kolkhir P Metz M Maurer M. The role and relevance of mast cells in urticaria. Immunol Rev. (2018) 282:232–47. 10.1111/imr.12632 29431202

[20] Kaplan AP Garofalo J Sigler R Hauber T. Idiopathic cold urticaria: *in vitro* demonstration of histamine release upon challenge of skin biopsies. N Engl J Med. (1981) 305:1074–7. 10.1056/NEJM198110293051808 6168912

[21] Kaplan AP. The pathogenic basis of urticaria and angioedema: recent advances. Am J Med. (1981) 70:755–8. 10.1016/0002-9343(81)90528-3 7211911

[22] Maurer M Metz M Brehler R Hillen U Jakob T Mahler V Omalizumab treatment in patients with chronic inducible urticaria: a systematic review of published evidence. J Allergy Clin Immunol. (2018) 141:638–49. 10.1016/j.jaci.2017.06.032 28751232

[23] Tanaka S Furuta K. Roles of IgE and histamine in mast cell maturation. Cells. (2021) 10:2170. 10.3390/cells10082170 34440939 PMC8392195

[24] Gimenez-Arnau AM Ribas-Llaurado C Mohammad-Porras N Deza G Pujol RM Gimeno R. Ige and high-affinity IgE receptor in chronic inducible urticaria, pathogenic, and management relevance. Clin Transl Allergy. (2022) 12:e12117. 10.1002/clt2.12117 35126995 PMC8805593

[25] Kolkhir P Altrichter S Hawro T Maurer M. C-reactive protein is linked to disease activity, impact, and response to treatment in patients with chronic spontaneous urticaria. Allergy. (2018) 73:940–8. 10.1111/all.13352 29130488

[26] Bizjak M Kosnik M Asero R Kocaturk E Gimenez-Arnau AM Maurer M. Lymphopenia in chronic spontaneous urticaria is linked to basopenia and eosinopenia. Clin Exp Allergy. (2024). 10.1111/cea.14538 39077869

[27] de la Cruz-Ku G Chambergo-Michilot D Torres-Roman JS Rebaza P Pinto J Araujo J Neutrophil-to-lymphocyte ratio predicts early mortality in females with metastatic triple-negative breast cancer. PLoS One. (2020) 15:e0243447. 10.1371/journal.pone.0243447 33284847 PMC7721150

[28] Elcioglu ZC Errington L Metes B Sendama W Powell J Simpson AJ Pooled prevalence of lymphopenia in all-cause hospitalisations and association with infection: a systematic review and meta-analysis. BMC Infect Dis. (2023) 23:848. 10.1186/s12879-023-08845-1 38042792 PMC10693046

[29] Kolkhir P Church MK Altrichter S Skov PS Hawro T Frischbutter S Eosinopenia, in chronic spontaneous urticaria, is associated with high disease activity, autoimmunity, and poor response to treatment. J Allergy Clin Immunol Pract. (2020) 8:318–25.e5. 10.1016/j.jaip.2019.08.025 31472293

[30] Kolkhir P Kovalkova E Chernov A Danilycheva I Krause K Sauer M Autoimmune chronic spontaneous urticaria detection with IgG anti-TPO and total IgE. J Allergy Clin Immunol Pract. (2021) 9:4138–46.e8. 10.1016/j.jaip.2021.07.043 34363991

[31] Ertas R Ozyurt K Ozlu E Ulas Y Avci A Atasoy M Increased IgE levels are linked to faster relapse in patients with omalizumab-discontinued chronic spontaneous urticaria. J Allergy Clin Immunol. (2017) 140:1749–51. 10.1016/j.jaci.2017.08.007 28870460

[32] Kolkhir P Laires PA Salameh P Asero R Bizjak M Kosnik M The benefit of complete response to treatment in patients with chronic spontaneous urticaria-CURE results. J Allergy Clin Immunol Pract. (2023) 11:610–20.e5. 10.1016/j.jaip.2022.11.016 36481420

[33] Bizjak M Rutkowski K Asero R. Risk of anaphylaxis associated with cold urticaria. Curr Treat Options Allergy. (2024) 11:167–75. 10.1007/s40521-024-00366-9

[34] Buttgereit T Salameh P Sydorenko O Zuberbier T Metz M Weller K The 7-day recall period version of the urticaria control test-UCT7. J Allergy Clin Immunol. (2023) 152:1210–7.e14. 10.1016/j.jaci.2023.03.034 37210040

[35] Maurer M Weller K Bindslev-Jensen C Gimenez-Arnau A Bousquet PJ Bousquet J Unmet clinical needs in chronic spontaneous urticaria. A GA(2)LEN task force report. Allergy. (2011) 66:317–30. 10.1111/j.1398-9995.2010.02496.x 21083565

[36] Maurer M Hawro T Krause K Magerl M Metz M Siebenhaar F Diagnosis and treatment of chronic inducible urticaria. Allergy. (2019) 74:2550–3. 10.1111/all.13878 31102545

[37] Toubi E Vadasz Z. Predictive features associated with chronic spontaneous urticaria recurrence. J Dermatol. (2021) 48:1786–8. 10.1111/1346-8138.16119 34519085

[38] Maurer M Magerl M. Differences and similarities in the mechanisms and clinical expression of bradykinin-mediated vs. mast cell-mediated angioedema. Clin Rev Allergy Immunol. (2021) 61:40–9. 10.1007/s12016-021-08841-w 33534062 PMC8282544

[39] Bizjak M Kosnik M Dinevski D Thomsen SF Fomina D Borzova E Risk factors for systemic reactions in typical cold urticaria: results from the COLD-CE study. Allergy. (2022) 77:2185–99. 10.1111/all.15194 34862605

[40] Acer E Kaya Erdogan H Yuksel Canakci N Saracoglu ZN. The effect of omalizumab on hematological and inflammatory parameters in patients with chronic spontaneous urticaria. Cutan Ocul Toxicol. (2019) 38:5–8. 10.1080/15569527.2018.1495227 29969297

[41] Deza G Ricketti PA Gimenez-Arnau AM Casale TB. Emerging biomarkers and therapeutic pipelines for chronic spontaneous urticaria. J Allergy Clin Immunol Pract. (2018) 6:1108–17. 10.1016/j.jaip.2018.02.024 30033912

[42] Pyatilova P Hackler Y Aulenbacher F Asero R Bauer A Bizjak M Non-skin related symptoms are common in chronic spontaneous urticaria and linked to active and uncontrolled disease: results from the chronic urticaria registry. J Allergy Clin Immunol Pract. (2024) 12:1890–9.e3. 10.1016/j.jaip.2024.04.027 38670260

[43] Seo IH Lee YJ. Usefulness of complete blood count (CBC) to assess cardiovascular and metabolic diseases in clinical settings: a comprehensive literature review. Biomedicines. (2022) 10:2697. 10.3390/biomedicines10112697 36359216 PMC9687310

[44] Karaman S Turedi B. Neutrophil-lymphocyte ratio: a possible marker of remission in children with chronic spontaneous urticaria. Allergol Immunopathol (Madr). (2020) 48:290–4. 10.1016/j.aller.2019.11.007 32299644

[45] Ertas R Ozyurt K Karakukcu C Akkus MR Ozlu E Avci A Evaluation of platelet parameters and neutrophil/lymphocyte ratio during omalizumab treatment in patients with severe chronic spontaneous urticaria. Turk J Med Sci. (2018) 48:1255–62. 10.3906/sag-1803-87 30541255

[46] Onder S Ozturk M. How does omalizumab affect the immunoinflammatory response in patients with chronic spontaneous urticaria? Cutan Ocul Toxicol. (2020) 39:31–5. 10.1080/15569527.2019.1684316 31642341

[47] Tarkowski B Lawniczak J Tomaszewska K Kurowski M Zalewska-Janowska A. Chronic urticaria treatment with omalizumab-verification of NLR, PLR, SIRI and SII as biomarkers and predictors of treatment efficacy. J Clin Med. (2023) 12:2639. 10.3390/jcm12072639 37048721 PMC10095242

[48] Kasperska-Zajac A Sztylc J Machura E Jop G. Plasma IL-6 concentration correlates with clinical disease activity and serum C-reactive protein concentration in chronic urticaria patients. Clin Exp Allergy. (2011) 41:1386–91. 10.1111/j.1365-2222.2011.03789.x 21645137

[49] Kolkhir P Andre F Church MK Maurer M Metz M. Potential blood biomarkers in chronic spontaneous urticaria. Clin Exp Allergy. (2017) 47:19–36. 10.1111/cea.12870 27926978

[50] Pedersen NH Sorensen JA Ghazanfar MN Zhang DG Vestergaard C Thomsen SF. Biomarkers for monitoring treatment response of omalizumab in patients with chronic urticaria. Int J Mol Sci. (2023) 24:11328. 10.3390/ijms241411328 37511088 PMC10379579

[51] Puxeddu I Petrelli F Angelotti F Croia C Migliorini P. Biomarkers in chronic spontaneous urticaria: current targets and clinical implications. J Asthma Allergy. (2019) 12:285–95. 10.2147/JAA.S184986 31571935 PMC6759208

[52] Guillen-Aguinaga S Jauregui Presa I Aguinaga-Ontoso E Guillen-Grima F Ferrer M. Updosing nonsedating antihistamines in patients with chronic spontaneous urticaria: a systematic review and meta-analysis. Br J Dermatol. (2016) 175:1153–65. 10.1111/bjd.14768 27237730

[53] Kolkhir P Borzova E Grattan C Asero R Pogorelov D Maurer M. Autoimmune comorbidity in chronic spontaneous urticaria: a systematic review. Autoimmun Rev. (2017) 16:1196–208. 10.1016/j.autrev.2017.10.003 29037900

[54] Zhang L Qiu L Wu J Qi Y Gao X He C GWAS of chronic spontaneous urticaria reveals genetic overlap with autoimmune diseases, not atopic diseases. J Invest Dermatol. (2023) 143:67–77.e15. 10.1016/j.jid.2022.07.012 35933036

[55] Neittaanmäki H. Cold urticaria. Clinical findings in 220 patients. J Am Acad Dermatol. (1985) 13:636–44. 10.1016/S0190-9622(85)70208-3 4078052

[56] Maltseva N Borzova E Fomina D Bizjak M Terhorst-Molawi D Kosnik M Cold urticaria—what we know and what we do not know. Allergy. (2021) 76:1077–94. 10.1111/all.14674 33249577

[57] Altrichter S Koch K Church MK Maurer M. Atopic predisposition in cholinergic urticaria patients and its implications. J Eur Acad Dermatol Venereol. (2016) 30:2060–5. 10.1111/jdv.13765 27324252

[58] Altrichter S Fok JS Jiao Q Kolkhir P Pyatilova P Romero SM Total IgE as a marker for chronic spontaneous urticaria. Allergy Asthma Immunol Res. (2021) 13:206–18. 10.4168/aair.2021.13.2.206 33474856 PMC7840871

