# Supplementary material for: Key differences between chronic inducible and spontaneous urticaria
Source: Front Allergy. 2024 Oct 17;5:1487831. doi: 10.3389/falgy.2024.1487831 (PMC11524999; doi:10.3389/falgy.2024.1487831)
Supplement: Supplementary file 1 [file Table1.docx]

***Supplementary Material***

**Supplementary Table 1.** Characteristics of CSU patients with high compared to normal CRP.

| Parameter | Total  n=285 | High CRP (≥5 mg/L)  n=68 (23.9) | Normal CRP  n=217 (76.1) | *p*-value |
| --- | --- | --- | --- | --- |
| Laboratory parameters |  |  |  |  |
| CRP (mg/L) | 1.9 (0.9-4.9) | 9.0 (6.1-17.3) | 1.3 (0.7-4.0) | ^NA^ |
| High CRP (≥5 mg/L) | 68 (23.9) | ^NA^ | ^NA^ | ^NA^ |
| Leukocytes (x 10^9^/L) | 6.91 (5.76-8.27) | 7.60 (6.75-8.79)↑ | 6.65 (5.56-7.92) | **<0.001** |
| Neutrophils (x 10^9^/L) | 4.37 (3.44-5.39) | 4.96 (4.19-6.10)↑ | 4.15 (3.16-5.18) | **<0.001** |
| NLR | 2.35 (1.78-3.16) | 2.81 (2.13-3.68)↑ | 2.27 (1.70-2.91) | **<0.001** |
| High NLR (≥2.5) | 123 (43.2) | 40 (58.8)↑ | 83 (38.2) | **0.003** |
| Lymphocytes (x 10^9^/L) | 1.80 (1.49-2.21) | 1.84 (1.39-2.15) | 1.80 (1.50-2.22) | 0.716 |
| Lymphopenia (<1.5 x 10^9^/L) | 72 (25.3) | 19 (27.9) | 53 (24.4) | 0.632 |
| Monocytes (x 10^9^/L) | 0.46 (0.37-0.59) | 0.46 (0.36-0.55) | 0.47 (0.38-0.59) | 0.520 |
| Eosinophils (x 10^9^/L) | 0.13 (0.07-0.21) | 0.12 (0.07-0.23) | 0.14 (0.08-0.20) | 0.642 |
| Eosinopenia (<0.05 x 10^9^/L) | 43 (15.1) | 12 (17.6) | 31 (14.3) | 0.560 |
| Basophils (x 10^9^/L) | 0.03 (0.01-0.04) | 0.02 (0.01-0.04) | 0.03 (0.02-0.04) | 0.052 |
| Basopenia (<0.01 x 10^9^/L) | 38 (13.3) | 12 (17.6) | 26 (12.0) | 0.227 |
| Platelets (x 10^9^/L) | 262.0 (219.5-302.0) | 278.5 (228.3-315.3)↑ | 254.0 (213.5-294.0) | **0.011** |
| PLR | 143.5 (114.3-174.3) | 153.8 (123.7-187.1)↑ | 141.5 (113.3-168.3) | **0.044** |
| Erythrocytes (x 10^12^/L) | 4.70 (4.36-4.96) | 4.75 (4.42-5.08) | 4.65 (4.34-4.90) | 0.147 |
| Total IgE (IU/mL) | 60.5 (22.0-128.8), n=120 | 51.0 (26.0-114.0), n=31 | 73.0 (21.0-133.5), n=89 | 0.496 |
| Clinical characteristics |  |  |  |  |
| Painful joints | 25 (8.8) | 11 (16.2)↑ | 14 (6.5) | **0.024** |
| Uncontrolled despite 4-fold sgAHs^a^ | 36 (17.9), n=201 | 14 (28.0), n=50 | 22 (14.6), n=151 | 0.053 |
| Controlled with up to 4-fold sgAHs^a^ | 165 (82.1), n=201 | 36 (72.0), n=50 | 129 (85.4), n=151 | 0.053 |

Note: Categorical data are reported as n (i.e., number of patients with the outcome) and percentage (i.e., number of patients with the outcome/total number of patients in the group). Numerical data are reported as median (IQR). If data were not obtained in all patients, patient numbers are displayed as “n”. Fisher's Exact test was used for categorical variables and the Mann-Whitney U test for numerical variables. Statistically significant p-values are given in bold. Arrows (↑ and ↓) indicate a significantly higher or lower level/frequency of a parameter in patients with high CRP compared to those with normal CRP.

^a^Uncontrolled and controlled CSU were defined as UCT=0−12 and UCT=12−16, respectively.

Abbreviations: *CRP*, C-reactive protein; *CSU*, chronic spontaneous urticaria; *IgE*, serum immunoglobulin E; *IQR,* interquartile range; *^NA^*, not applicable; *NLR*, neutrophil-to-lymphocyte ratio; *PLR*, platelet-to-lymphocyte ratio; *sgAHs,* second-generation H_1_-antihistamines; *UCT*, Urticaria Control Test.
