# Supplementary material for: Key differences between chronic inducible and spontaneous urticaria
Source: Front Allergy. 2024 Oct 17;5:1487831. doi: 10.3389/falgy.2024.1487831 (PMC11524999; doi:10.3389/falgy.2024.1487831)
Supplement: Supplementary file 3 [file Table3.docx]

***Supplementary Material***

**Supplementary Table 3.** Parameters linked to response to standard treatment in CSU.

| Parameter | sgAHs taken daily  n=207 | Uncontrolled despite 4-fold^a^  n=37 (17.9) | Controlled with up to 4-fold^a^  n=170 (82.1) | *p*-value |
| --- | --- | --- | --- | --- |
| Female gender | 138 (66.7) | 29 (78.4) | 109 (64.1) | 0.124 |
| CRP (mg/L) | 1.9 (0.9-5.0), n=201 | 3.9 (0.8-12.0), n=36↑ | 1.7 (0.9-4.5), n=165 | **0.026** |
| Neutrophils (x 10^9^/L) | 4.33 (3.51-5.41) | 4.44 (3.80-5.40) | 4.33 (3.46-5.42) | 0.655 |
| NLR | 2.36 (1.78-3.17) | 2.35 (1.71-3.17) | 2.43 (1.96-3.33) | 0.490 |
| Lymphocytes (x 10^9^/L) | 1.81 (1.48-2.23) | 1.67 (1.39-2.04) | 1.85 (1.49-2.26) | 0.261 |
| Lymphopenia (<1.5 x 10^9^/L | 55 (26.6) | 11 (29.7) | 44 (25.9) | 0.682 |
| Monocytes (x 10^9^/L) | 0.46 (0.37-0.57) | 0.44 (0.34-0.55) | 0.47 (0.37-0.59) | 0.308 |
| Eosinophils (x 10^9^/L) | 0.12 (0.07-0.21) | 0.10 (0.06-0.16)↓ | 0.14 (0.07-0.22) | **0.033** |
| Eosinopenia (<0.05 x 10^9^/L) | 30 (14.5) | 7 (18.9) | 23 (13.5) | 0.440 |
| Basophils (x 10^9^/L) | 0.03 (0.01-0.04) | 0.01 (0-0.03) | 0.03 (0.02-0.04) | **<0.001** |
| Basopenia (<0.01 x 10^9^/L) | 32 (15.5) | 14 (37.8)↑ | 18 (10.6) | **<0.001** |
| Platelets (x 10^9^/L) | 266.0 (220-311.0) | 260.0 (228.0-312.0) | 266.0 (218.5-310.5) | 0.984 |
| PLR | 143.6 (112.3-176.3) | 149.2 (117.5-182.5) | 141.5 (110.6-176.2) | 0.336 |
| Total IgE (IU/mL) | 51.0 (13.8-111.0), n=84 | 56.0 (20.5-117.5), n=19 | 38.0 (4.0-90.0), n=65 | 0.196 |
| Parameter | **OMA treatment**  **(300 mg/4 weeks)**  **n=28** | **Uncontrolled in 12 weeks**^a^  **n=8 (28.6)** | **Controlled in 12 weeks**^a^  **n=20 (71.4)** | ***p*-value** |
| Female gender | 19 (67.9) | 7 (87.5) | 12 (60.0) | 0.214 |
| CRP (mg/L) | 2.0 (0.7-4.7) | 2.3 (0.7-6.2) | 1.8 (0.7-4.7) | 0.618 |
| Neutrophils (x 10^9^/L) | 4.20 (3.63-4.90) | 3.95 (3.63-4.47) | 4.34 (3.53-5.83) | 0.263 |
| NLR | 2.47 (1.96-3.46) | 2.47 (1.81-3.43) | 2.87 (2.17-3.59) | 0.476 |
| Lymphocytes (x 10^9^/L) | 1.67 (1.29-2.06) | 1.48 (1.09-1.81) | 1.74 (1.55-2.38) | 0.071 |
| Lymphopenia (<1.5 x 10^9^/L | 8 (28.6) | 4 (50.0) | 4 (20.0) | 0.172 |
| Monocytes (x 10^9^/L) | 0.44 (0.33-0.55) | 0.34 (0.26-0.43)↓ | 0.45 (0.35-0.71) | **0.032** |
| Eosinophils (x 10^9^/L) | 0.12 (0.08-0.21) | 0.10 (0.07-0.11)↓ | 0.17 (0.09-0.24) | **0.049** |
| Eosinopenia (<0.05 x 10^9^/L) | 3 (10.7) | 1 (12.5) | 2 (10.0) | 1.000 |
| Basophils (x 10^9^/L) | 0.02 (0.01-0.04) | 0.01 (0-0.03) | 0.02 (0.01-0.04) | 0.056 |
| Basopenia (<0.01 x 10^9^/L) | 8 (28.6) | 5 (62.5)↑ | 3 (15.0) | **0.022** |
| Platelets (x 10^9^/L) | 246.5 (221.3-314.5) | 275.0 (207.3-314.5) | 246.5 (234.5-327.8) | 0.939 |
| PLR | 154.1 (127.4-189.2) | 188.6 (161.8-217.9)↑ | 144.3 (119.2-165.2) | **0.006** |
| Total IgE (IU/mL) | 34.0 (2.5-73.5), n=13 | 58.0 (1.0-117.0), n=6 | 21.0 (2.8-57.0), n=7 | 0.567 |
| Parameter | **Controlled with OMA**  **in 12 weeks^a^**  **n=20** | **Late response**  **(by week 12)**  **n=9 (45.0)** | **Fast response**  **(by week 4)**  **n=11 (55.0)** | ***p*-value** |
| Female gender | 12 (60.0) | 8 (88.9)↑ | 4 (36.4) | **0.028** |
| CRP (mg/L) | 1.8 (0.7-4.7) | 4.6 (1.6-8.7)↑ | 0.9 (0.5-2.2) | **0.044** |
| Neutrophils (x 10^9^/L) | 4.34 (3.53-5.83) | 4.55 (4.00-6.29) | 4.15 (2.94-4.95) | 0.171 |
| NLR | 2.87 (2.17-3.59) | 2.75 (1.92-3.40) | 2.22 (1.69-3.47) | 0.621 |
| Lymphocytes (x 10^9^/L) | 1.74 (1.55-2.38) | 1.79 (1.41-2.65) | 1.67 (1.57-2.06) | 0.543 |
| Lymphopenia (<1.5 x 10^9^/L | 4 (20.0) | 2 (22.2) | 2 (18.2) | 1.000 |
| Monocytes (x 10^9^/L) | 0.45 (0.35-0.71) | 0.74 (0.41-0.87)↑ | 0.43 (0.33-.052) | **0.020** |
| Eosinophils (x 10^9^/L) | 0.17 (0.09-0.24) | 0.22 (0.11-0.29) | 0.13 (0.07-0.19) | 0.159 |
| Eosinopenia (<0.05 x 10^9^/L) | 2 (10.0) | 0 | 2 (18.2) | 0.479 |
| Basophils (x 10^9^/L) | 0.02 (0.01-0.04) | 0.02 (0.01-0.04) | 0.02 (0.01-0.04) | 0.352 |
| Basopenia (<0.01 x 10^9^/L) | 3 (15.0) | 3 (33.3) | 0 | 0.074 |
| Platelets (x 10^9^/L) | 246.5 (234.5-327.8) | 252.0 (235.0-346.5) | 242.0 (217.0-309.0) | 0.543 |
| PLR | 144.3 (119.2-165.2) | 140.8 (113.0-178.6) | 146.7 (120.4-165.0) | 0.732 |
| Total IgE (IU/mL) | 21.0 (2.8-57.0), n=7 | 29.5 (1.0-87.5), n=4 | 72.0 (52.0-169.5), n=3 | 0.285 |

Note: This table presents a comparison between responders versus non-responders to sgAHs, responders versus non-responders to OMA, and fast (i.e., by week 4) versus late (i.e., by week 12) responders to OMA. Categorical data are reported as n (i.e., number of patients with the outcome) and percentage (i.e., number of patients with the outcome/total number of patients in the group). Numerical data are reported as median (IQR). Fisher's Exact test was used for categorical variables and the Mann-Whitney U test for numerical variables. Statistically significant p-values are given in bold.

^a^Uncontrolled and controlled CSU were defined as UCT=0−12 and UCT=12−16, respectively.

Arrows (↑ and ↓) indicate a significantly higher or lower level/frequency of a parameter in a group in the 3^rd^ column compared to the group in the 4^th^ column.

Abbreviations: *CRP*, C-reactive protein; *CSU-alone*, chronic spontaneous urticaria without concomitant chronic inducible urticaria; *IgE*, serum immunoglobulin E; *IQR,* interquartile range; *NLR*, neutrophil-to-lymphocyte ratio; *OMA*, omalizumab; *PLR*, platelet-to-lymphocyte ratio; *sgAHs,* second-generation H_1_-antihistamines; *UCT,* Urticaria Control Test.
